# Opposing MMP-9 Expression in Mesenchymal Stromal Cells and Head and Neck Tumor Cells after Direct 2D and 3D Co-Culture

**DOI:** 10.3390/ijms24021293

**Published:** 2023-01-09

**Authors:** Anna Waltera, Daniela Schulz, Nicole Schaefer, Sabine Stoeckl, Eric Pion, Silke Haerteis, Torsten E. Reichert, Tobias Ettl, Richard J. Bauer

**Affiliations:** 1 Department of Oral and Maxillofacial Surgery, University Hospital Regensburg, 93053 Regensburg, Germany; 2 Department of Oral and Maxillofacial Surgery, Center for Medical Biotechnology, University Hospital Regensburg, 93053 Regensburg, Germany; 3 Experimental Orthopaedics, Centre for Medical Biotechnology (ZMB), Bio Park 1, University of Regensburg, 93053 Regensburg, Germany; 4 Institute for Molecular and Cellular Anatomy, University of Regensburg, 93053 Regensburg, Germany

**Keywords:** HNSCC, head and neck cancer, BMSC, bone marrow-derived stromal cells, angiogenesis, MMP, matrix metalloproteinase, CAM, chorioallantoic membrane, in ovo, 2D, 3D

## Abstract

Bone marrow-derived mesenchymal stromal cells (BMSCs) respond to a variety of tumor cell-derived signals, such as inflammatory cytokines and growth factors. As a result, the inflammatory tumor microenvironment may lead to the recruitment of BMSCs. Whether BMSCs in the tumor environment are more likely to promote tumor growth or tumor suppression is still controversial. In our experiments, direct 3D co-culture of BMSCs with tumor cells from the head and neck region (HNSCC) results in strong expression and secretion of MMP-9. The observed MMP-9 secretion mainly originates from BMSCs, leading to increased invasiveness. In addition to our in vitro data, we show in vivo data based on the chorioallantoic membrane (CAM) model. Our results demonstrate that MMP-9 induces hemorrhage and increased perfusion in BMSC/HNSCC co-culture. While we had previously outlined that MMP-9 expression and secretion originate from BMSCs, our data showed a strong downregulation of MMP-9 promoter activity in HNSCC cells upon direct contact with BMSCs using the luciferase activity assay. Interestingly, the 2D and 3D models of direct co-culture suggest different drivers for the downregulation of MMP-9 promoter activity. Whereas the 3D model depicts a BMSC-dependent downregulation, the 2D model shows cell density-dependent downregulation. In summary, our data suggest that the direct interaction of HNSCC cells and BMSCs promotes tumor progression by significantly facilitating angiogenesis via MMP-9 expression. On the other hand, data from 3D and 2D co-culture models indicate opposing regulation of the MMP-9 promoter in tumor cells once stromal cells are involved.

## 1. Introduction

Head and neck squamous cell carcinoma (HNSCC) is the seventh most common cancer worldwide, with a steady increase in incidence over the past 10 years [1]. Unfortunately, despite all medical advances in recent decades, the 5-year survival rate is still only 50–60% [2]. Besides the difficult treatability of HPV-negative head and neck cancer, other reasons for the high mortality rate may be the often late diagnosis at advanced stages and the heterogeneity within head and neck tumors [3,4]. The main risk factors for developing head and neck cancer are tobacco and alcohol abuse [5], which explains why most head and neck tumors occur in the oral cavity [6,7]. Cancer in the oral cavity, in particular, spreads rapidly and invasively to surrounding anatomic structures, including lymph nodes. Therefore, local recurrences in the cervical region or remotely are relatively common. More than 60% of patients have an advanced stage at initial diagnosis, in which adjacent tissues and lymph nodes are already infiltrated. Bone invasion can also be observed in approximately 20% of HNSCC patients [8,9,10]. From a tumor biology perspective, it remains elusive why some oral floor carcinomas grow rapidly and invasively while others grow but do not infiltrate adjacent tissues.

The molecular mechanisms of cell invasion are now well understood. Nevertheless, the interaction with the surrounding tumor stroma and stromal cells seems to play a fundamental role. Therefore, current research focuses not only on the tumor cells themselves but also on the tumor microenvironment, as it is crucial for tumor progression. In this context, Bone marrow-derived mesenchymal stromal cells (BMSCs) are of particular interest [11,12].

BMSCs can differentiate into chondrogenic, osteogenic, and adipogenic cell lineages depending on their location and extracellular stimuli [13]. Physiologically, BMSCs are involved in wound healing, hematopoiesis and tissue regeneration, for instance. Furthermore, BMSCs can also be detected in tumor tissue [14,15]. Along with fibroblasts and various inflammatory cells, BMSCs are recruited to the tumor microenvironment in response to various signals from tumor cells, such as inflammatory cytokines and growth factors [16,17]. Circulating BMSCs settle in the tumor stroma or are incorporated into the tumor mass [18]. However, their function in this context has not been fully elucidated. Currently, there is controversial literature attributing both tumor-promoting and tumor-inhibiting properties to BMSCs [19,20]. Depending on the tumor type and tissue, several studies and clinical trials have reported opposing effects of transplanted BMSCs on tumor metabolism. Most research currently supports that infiltration of MSCs promotes the development of more aggressive tumor characteristics [21,22,23,24,25,26,27,28,29]. Some studies show an inhibitory effect on cancer cell growth and metastasis [30,31,32,33].

Interestingly, osteogenic differentiation markers of tumor microenvironment BMSCs correlate with poorer prognosis in this regard. Our results serve to further elucidate the effects of BMSCs on HNSCC cells. We focused on the expression of matrix metalloproteases (MMPs), particularly MMP-9, a key protein in tumor progression that facilitates migration and invasion, thereby promoting tumor metastasis [34,35]. MMPs belong to a large family of zinc endopeptidases that can cleave specific extracellular matrices. Currently, a total of 28 human MMPs have been discovered [36,37,38]. MMP-1, MMP-2, and MMP-9 are among the most frequently mutated MMPs in OSCC and facilitate invasiveness and metastasis to adjacent tissue structures [39,40]. In a previous publication, we demonstrated that 3D spheroid co-culture of BMSCs and HNSCC cells results in differential expression and secretion of MMP-9 as well as induction of osteogenic markers such as ALP and Runx2. MMP-9 is generally associated with increased invasion, metastasis, and progression of a wide variety of tumor entities, including HNSCC [34,41].

The observation that infiltration of BMSCs promotes the development of more aggressive tumor characteristics has also been shown in our own work [42]. The exact cause of the increased production of MMP-9 in tumor tissue is poorly understood to date. The aim of this work was to further elucidate the effects of the interaction of BMSCs and tumor cells of the head and neck region. To this end, starting from HNSCC cell lines in co-culture with BMSCs, the effect on protein expression of MMP-9 was investigated. In addition, this work served to further investigate MMP-9 promoter activity in HNSCC cells and BMSCs. The secretion and activity of proteases triggered by the interaction of different cell types can lead to shifts in the fine balance of matrix remodeling. Remodeled matrix environments can become targets for aberrant cells. As a result, these cells can migrate and invade more easily due to the disruption of matrix arrangement and structure. MMP-9 has been shown to be induced in heterogeneous spheroid co-cultures of BMSCs and HNSCC cells [42]. Here, we show additional data from the chorioallantoic membrane (CAM) model supporting these data. We also demonstrated that MMP-9 induction originates from BMSCs through direct cell contact with tumor cells. We were now interested in understanding how MMP-9 expression changes in HNSCC cells when they come into contact with BMSCs. In this manuscript, we take a closer look at the promoter activity of MMP-9 in HNSCC cells. We also highlight the differences we observed in 2D and 3D cell cultures.

## 2. Results

### 2.1. BMSC/HNSCC Co-Culture Increases Neoangiogenesis and Increased Perfusion in the CAM Model

**Figure 1:**
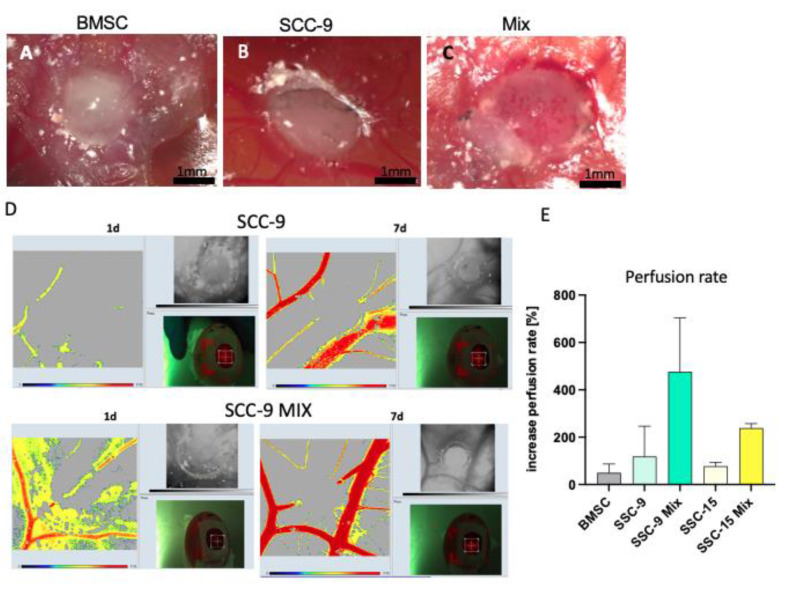
(**A**–**C**): An exemplified image of BMSC, HNSCC (SCC-9), and BMSC/HNSCC co-culture 3D spheroids (mix) placed on the chorioallantoic membrane (CAM) of a chicken egg. After 7 days, all heterogeneous co-cultures showed markedly increased neoangiogenesis and visible ingrowing blood vessels. In contrast, the homogeneous spheroids did not exhibit neoangiogenesis or ingrowing blood vessels. (**D**): Exemplified images of LSCI measurements. Measurement of perfusion revealed greatly increased blood flow in the co-cultures within 7 days (**D**,**E**). *n* = 3. HNSCC: head and neck squamous cell carcinoma cell lines, BMSC: bone marrow-derived stromal cells, mix: co-culture of BMSCs and HNSCC cells. Bar = 1 mm.

Wessely et al. demonstrated that the gene expression, secretion, and activity of MMP-9 were exclusively increased in BMSC/HNSCC co-culture but not in mono-culture [42]. This was due to the induction of MMP-9 expression in BMSCs after contact with tumor cells. Here, we confirm and supplement these data with results obtained in the chicken chorioallantoic membrane (CAM) model with two additional cell lines, SCC-9 and SCC-15. After 7 days, all co-culture mixtures of 4000 BMSCs with 18,000 SCC-9 cells showed visible ingrowth of blood vessels and blood leakage (Figure 1C). In contrast, the mono-culture 3D spheroids (22,000 cells) exhibited no observable ingrowth of blood vessels (Figure 1A,B). The in ovo measurement of perfusion rate by laser speckle contrast imaging (LSCI) showed that blood flow and vascular sprouting were greatly increased in the mixed cultures compared with the mono-cultures from day 1 to day 7 (Figure 1D,E).

### 2.2. BMSC/HNSCC Co-Culture Increases Expression and Secretion of MMP-9

**Figure 2:**
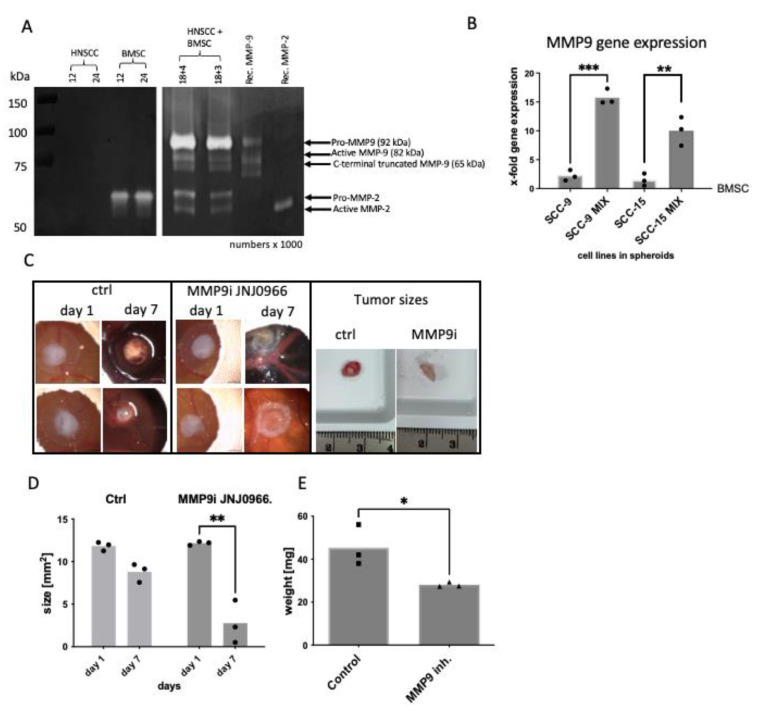
(**A**): Exemplary zymography depicting the exclusive expression and secretion of MMP-9 in the BMSC/HNSCC co-culture 3D spheroids (mix) compared to HNSCC and BMSC mono-culture 3D spheroids. Left panel: Supernatants of HNSCC cells and BMSC mono-culture 3D spheroids with 12,000 or 24,000 cells, respectively. Right panel: BMSC/HNSCC co-culture with 18,000 tumor cells each and 4000 or 3000 BMSCs. With 3000 BMSCs, the detection of active MMP-9 decreases. The pro-form of MMP-9 is strongly expressed in all mixtures. (**B**): Taqman quantitative PCR revealed that MMP-9 was also significantly increased in the mixture compared with the mono-cultures in the CAM model. (**C**–**E**): Specific MMP-9 inhibitor (MMP9i, JNJ0966, Selleckchem, Houston, TX, USA) stopped the severe hemorrhage of the mixtures and significantly reduced tumor size and weight in the CAM model (see also Supplementary Figure S1). *n* = 3, numbers × 1000. HNSCC: head and neck squamous cell carcinoma cell lines, BMSC: bone marrow-derived stromal cells, Rec. MMP: recombinant MMP controls. Unpaired *t*-test: * *p* < 0.05; ** *p* < 0.01; *** *p* < 0.001.

Additionally, zymography demonstrates the exclusive expression and secretion of the inactive pro-enzyme and active/truncated forms of MMP-9 in BMSC/HNSCC co-culture with two different BMSC/HNSCC ratios (Figure 2A, right panel). 3D mono-cultured BMSC spheroids secrete merely the inactive pro-enzyme of MMP-2 (66 kDa) and no MMP-9, whereas mono-culture tumor spheroids show very little or no expression and secretion of MMP-2 and MMP-9 (Figure 2A). This has been shown by us in Wessely et al. [42]. Here, using two head and neck tumor cell lines, SCC-9 and SCC-15, we confirmed increased MMP-9 gene expression in co-culture 3D spheroids compared to mono-culture 3D spheroids on the CAM model using quantitative Taqman PCR (Figure 2B).

### 2.3. MMP-9 Secretion and Activation Are Responsible for Hemorrhage in the CAM Model

We further investigated whether the strong MMP-9 expression and secretion in the co-culture were involved in the development of severe hemorrhages. After targeting MMP-9 with the specific MMP-9 inhibitor JNJ0966 (Selleckchem, Houston, TX, USA), which blocks the conversion of pro-MMP-9 to active MMP-9, we observed a significant decrease in tumor size after 7 days of incubation on the CAM compared with day 1 (d1: 12.15 µm^2^ vs. d7: 2.8 µm^2^ mean value, with a mean d1–d7 difference of 9.35 µm^2^). There was no significant difference in the control samples treated with appropriate concentration of the solvent DMSO (d1: 11.82 µm^2^ vs. d7: 8.8 µm^2^ mean value, with a mean d1–d7 difference of 3 µm^2^, Figure 2C,D, Supplementary Figure S1). The weight of the control samples after 7 days of growth on the CAM without treatment was higher (45.4 mg, mean value) compared to the weight of treated samples (28.1 mg, mean value, Figure 2E). In addition, it was evident that there was no further hemorrhage after MMP-9 inhibition (Figure 2C, Supplementary Figure S1).

### 2.4. MMP-9 Promotor Activity in HNSCC Cells Differs Significantly in 2D and 3D Cell Culture

We have formerly demonstrated that the increased MMP-9 expression originates from BMSCs and not from tumor cells [42]. Nevertheless, we wanted to elucidate if the MMP-9 promoter in HNSCC cells was active and to what extent. For this purpose, we generated a luciferase construct with an approximately 960 bp long promoter sequence of MMP-9 and transfected the HNSCC cell lines PCI 1, PCI 13 and PCI 68. Simultaneously, we determined whether differences occur in 2D and 3D cell culture models.

**Figure 3:**
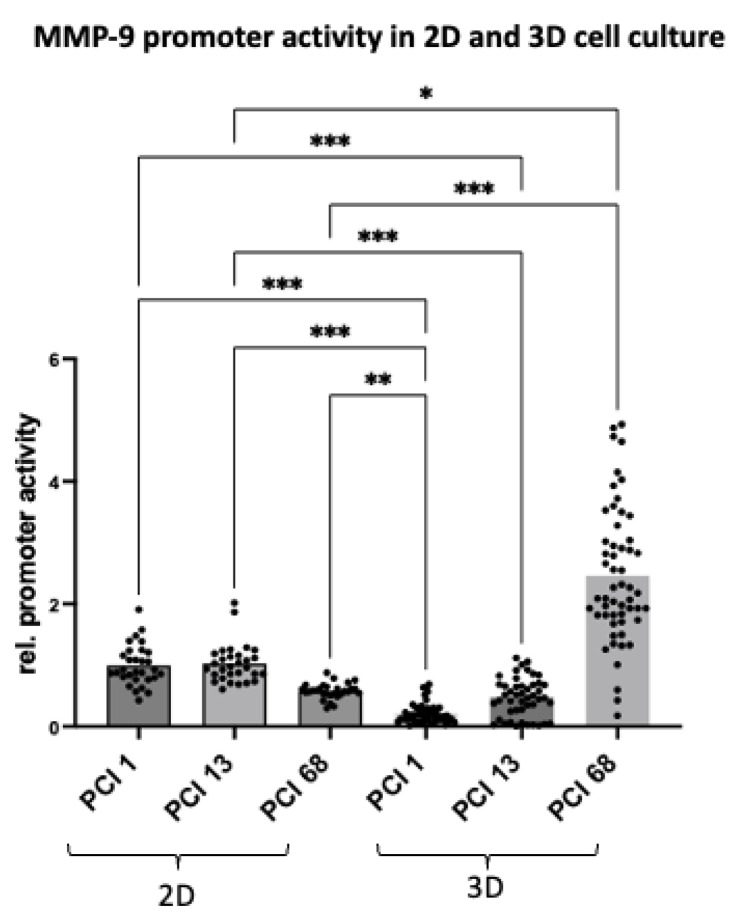
Relative MMP-9 promoter activity in HNSCC cells from 2D and 3D cell cultivation by means of luciferase activity assay. Significant differences can be observed in the MMP-9 promoter activity in the 2D and 3D models. Except for PCI 68, HNSCC cells reveal lower basal MMP-9 promoter activity in 3D spheroid culture. Promoter activity in 2D and 3D cell culture was calculated relative to promoter activity in HNSCC cell line PCI 1 from adherent 2D cell culture. For 3D cell culture, spheroids were cultured for three days. Statistical analysis: ordinary one-way ANOVA, multiple comparisons with Tukey’s correction. * *p* < 0.05; ** *p* < 0.01; *** *p* < 0.001. *n* ≥ 30.

Figure 3 shows detectable MMP-9 promoter activity in all examined tumor cell lines examined. Basal activity differed between the 2D and 3D culture models as well as between HNSCC cell lines.

In adherent 2D cell culture, PCI 1 and PCI 13 exhibited comparable MMP-9 promoter activity with minor differences. Interestingly, PCI 68 revealed a significant reduction of MMP-9 promoter activity by 42% compared with cell lines PCI 1 and PCI 13.

When cultured in 3D spheroids, there was a significant decrease in promoter activity in PCI 1 (78%) and PCI 13 (52%) compared to the adherent 2D cell culture. In contrast, activity in PCI 68 increased 4.2-fold and showed the highest MMP-9 promoter activity among the three HNSCC cell lines examined (Figure 3).

### 2.5. Decrease of MMP-9 Promoter Activity in HNSCC Cells When Co-Cultured with BMSCs in 2D and 3D

**Figure 4:**
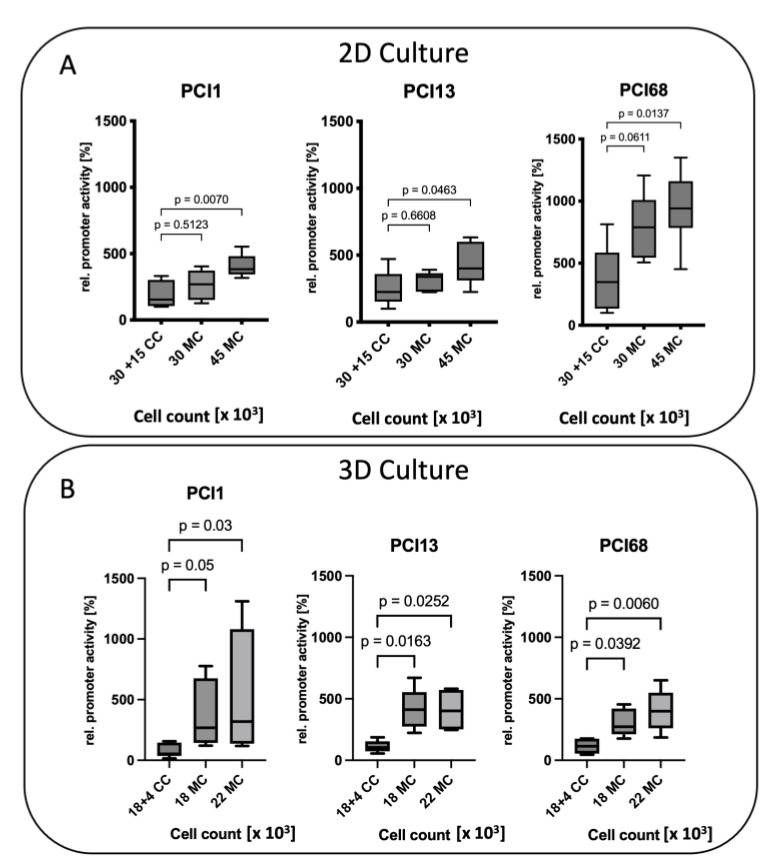
Decrease of MMP-9 promoter activity of HNSCC cells when co-cultured with BMSCs in direct 2D (**A**) and 3D co-culture (**B**). The relative MMP-9 promoter activity of HNSCC cell lines PCI 1, PCI 13 and PCI 68 when co-cultured with BMSCs (CC) was calculated. Their activity was compared with the MMP-9 promoter activity of HNSCC cells in mono-culture (MC). Relative promoter activity was calculated in relation to the mean value of activity from 2D/3D CC of the respective cell lines. Cell count × 1000. Statistical analysis: One way ANOVA. *n* = 5–6.

To investigate whether the MMP-9 promoter activity in HNSCC cells changed upon contact with BMSCs, BMSC/HNSCC co-cultures (CC) were analyzed in comparison with HNSCC cells in mono-culture (MC). The experiments were performed in both the 2D and 3D models (Figure 4), whereby HNSCC cell lines were transfected with the luciferase reporter but not the BMSCs.

In the adherent 2D culture, MMP-9 promoter activity does not change considerably if CC (30,000 HNSCCs + 15,000 BMSCs) are compared with an equal amount of MC tumor cells (30,000 HNSCCs) in PCI 1 and PCI 13. Only when cell numbers were increased in MC to an equal overall amount (45,000 cells) the promoter activity increased significantly in the MC in all three cell lines (Figure 4A).

Interestingly, in 3D spheroid cell culture, cell number does not seem to play as important a role as in adherent 2D culture. In spheroids, the presence of BMSCs appears to play a major role in downregulating the MMP-9 promoter in the tumor cells. Here, all three HNSCC cell lines showed significant 5–10-fold downregulation of MMP-9 promoter activity in CC with BMSCs compared with MC regardless of the respective cell number or cell density (Figure 4B).

### 2.6. Influence of Cell Density on MMP-9 Promoter Activity in 2D and 3D

**Figure 5:**
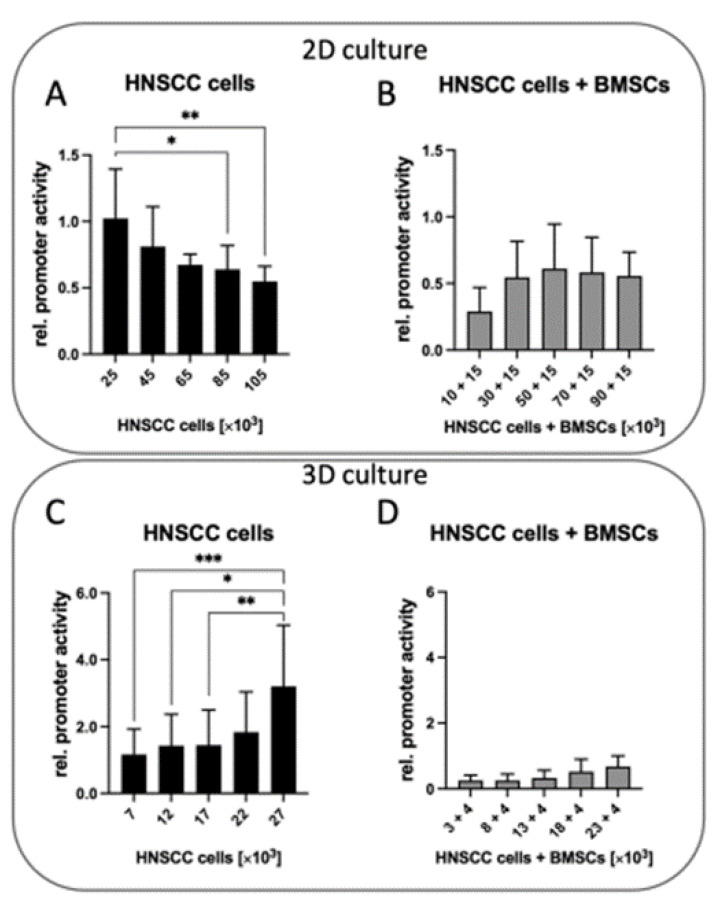
Pooled analysis of MMP-9 promoter activity in three HNSCC cell lines PCI 1, 13 and 68. (**A**,**B**): MMP-9 promoter activity in adherent 2D cell culture. (**A**): The mono-culture of HNSCC cells exhibits decreasing MMP-9 promoter activity with increasing cell density. (**B**): A mixture of HNSCC cells with BMSCs results in equal promoter activity regardless of the cell number. (**C**,**D**): Promoter activity in 3D spheroid culture. (**C**): MMP-9 promoter activity in HNSCC cells increases significantly in proportion to cell count. (**D**): Overall, significant attenuation of MMP-9 promoter activity in HNSCC/BMSC mixtures compared to mono-culture spheroids. There is only a slight increase in MMP-9 promoter activity with rising cell counts. * *p* < 0.05; ** *p* < 0.01; *** *p* < 0.001. *n* = 9.

We additionally analyzed the influence of cell density on MMP-9 promoter activity in 2D and 3D. Pooled Analysis of HNSCC cells in adherent 2D mono-culture showed an effect of confluence on MMP-9 promoter activity. Figure 5A illustrates a decrease in MMP-9 promoter activity with an increasing initial seeded cell number. At very high confluence, with 85,000 and 105,000 HNSCC cells, this observation proved significant (*p* = 0.02 and *p* = 0.002, respectively).

In HNSCC/BMSC co-cultures, increasing the number of HNSCC cells while keeping the number of BMSCs constant had no significant effect on promoter activity (Figure 5B). This appeared entirely reversed in 3D spheroid culture. Overall, compared to the 3D monoculture spheroids, all the mixing ratios exhibited a strong downregulation of MMP-9 promoter activity in HNSCC cells (Figure 5D). 3D HNSCC cell mono-culture revealed cell number-dependent 4–5-fold increase in MMP-9 promoter activity (Figure 5C). Figure 5C shows spheroid cell count having a significant effect on MMP-9 promoter activity in 3D MC HNSCC cells. Here, the MMP-9 promoter activity increased significantly in proportion to the cell number. In contrast, when tumor cells are 3D co-cultured with BMSCs, there was only a small increase in MMP-9 promoter activity in tumor cells depending on the cell number (Figure 5D).

## 3. Discussion

For a long time, mesenchymal stem/stromal cells (MSCs) have been the subject of intensive research [24]. MSCs are highly motile. They promote tissue regeneration and wound healing in physiologically healthy organisms. Oncological research gained interest in MSCs due to their migratory abilities to invade tumor entities [43,44]. Here, both tumor-promoting and tumor-inhibiting effects of MSCs were found [28]. It is becoming increasingly clear that the interplay between cancer cells and cells of the stroma is involved in the acquired capacity for invasive growth and metastasis. This signaling can act on cancerous cells to alter their characteristic capabilities [45]. For instance, MSCs in the tumor stroma, in response to signals released by cancer cells, were found to secrete CCL5/RANTES; CCL5 then acts inversely on cancer cells and stimulates invasive behavior [46].

One of our main observations so far is that after mixing BMSCs from different donors with different HNSCC cell lines, besides the increased ALP activity, a strong differential upregulation of MMP-9 expression and secretion was observable. This was accompanied by a strongly increased invasion ability of the co-culture. The effect occurred exclusively in all co-cultures, but mono-cultures showed little MMP-9 expression [42]. In this study, we used the CAM model to demonstrate that the co-culture of tumor cells with BMSCs clearly promotes blood vessel growth and angiogenesis. This was demonstrated visually and quantitively via perfusion measurement.

The CAM model is a powerful in vivo tumor model [47,48,49,50]. A tumor mass can form a few days after transplantation, which is very similar to the original tumor [51]. This rapid formation of tumor mass is due to the nutrient-rich environment of the CAM. An important achievement of the CAM model is that patient tumor samples can be used for tissue engraftment onto the CAM, allowing for the formation of a patient-derived CAM tumor [48,52]. To measure perfusion within 7 days, we used the laser-speckled contrast imaging system (LSCI). Pion et al. and Mena Kuri et al. demonstrated this system to be an easy-to-perform method to study blood flow rate in the CAM model [53].

MMP-9 is a member of a large family of matrix remodeling proteases. It is expressed in invasive tumors and highly involved in tumor progression, whereas in normal cells, MMP-9 expression is negligible [54]. Many of these proteases are involved in tumor growth and angiogenesis [41,55,56,57]. This means they enable the invasion and migration of cells but also, as in the case of MMP-9, angiogenesis [56,57,58,59].

In our group, we have shown MMP-9 to be one critical protease for head and neck cancer invasion [42,60]. Other groups have corroborated the data, showing that treatment with RNA interference or antigelatinolytic peptides against MMP-9 affected the invasive behavior of OSCC and ovarian cancer cells [61,62]. The supernatants of 3D spheroid mono-cultures showed very reduced or no MMP-9 secretion and activity, although promoter activity seems to vary depending on cell density and contact with stromal cells. In cell lysates of mono-cultures, we did observe minor expression levels of MMP-9. Therefore, our notion is that HNSCC cells need an additional external stimulus, for example, stromal cells or specific matrix components in the tumor microenvironment, for MMP-9 secretion and activation.

In human skin keratinocytes, dermal fibroblasts, and rat hepatic stellate cells, TNFalpha induces MMP-9 via p21-activated kinase-1. In this context, activation of Jun N-terminal kinase (JNK) and NF-kappaB was essential for MMP-9 activation [63]. Our MMP-9 luc-reporter contains two AP-1 binding sites. With this reporter, we have demonstrated that MMP-9 expression can be modulated via differential interaction and localization of AP-1 transcription factors like c-Fos and JunB upon collagen type XVI induction [60]. This could also be a regulatory mechanism in tumor cell mono-cultures. Interaction with BMSCs could alter the amount of AP-1 transcription factors, which is reflected in the downregulation of promoter activity.

In previous studies, we have shown that the stimulus of collagen type XVI triggers MMP-9 secretion in HNSCC cells, leading to enhanced invasion. It was demonstrated that MMP-9 secretion and activation could be induced via integrin-linked kinase activation. In the absence of either of these external stimuli, MMP-9 secretion and activation may be restrained [60,64]. Moreover, promoter activity is not necessarily related to protein secretion and activation. Especially since the secretion and activation of MMPs are tightly controlled [65]. The interaction and regulation by proteins like tissue inhibitors of matrix metalloproteinases (TIMPs) add to the complexity of the expression and secretion mechanism of MMPs. Roderfeld and colleagues, for example, demonstrated that the MMP-9 pro-form is already present as a complex with TIMP-1 in the Golgi apparatus before secretion [66]. Further studies definitely have to address the exact mechanism of how MMP-9 secretion in HNSCC cells is controlled.

MMP-9 in head and neck carcinoma is associated with shortened relapse-free and cause-specific survival, implying that MMP-9 has a role in tumor progression of head and neck carcinomas [67]. Furthermore, MMP-9 is a predictor of tumor recurrence in oral squamous cell carcinoma [68]. Literature shows the involvement of MMP-9 in tumor angiogenesis. Bergers et al. were able to show that MMP-9 is crucial in the activation and recruitment of new blood vessels during the development of tumors in pancreatic islets. They demonstrated that active MMP-9 is selectively able to transform normal non-angiogenic islets into angiogenic islets by releasing VEGF [59]. Moreover, decreased expression of VEGF and MMP-9 in medulloblastoma cells overexpressing osteonectin resulted in decreased angiogenesis and tumor growth, indicating the pro-angiogenic role of MMP-9 in cancer tissues [69]. In addition, a cooperative effect of MMP-2 and MMP-9 was demonstrated in an experimental in vivo model, which enhanced the angiogenic phenotype and invasiveness of tumor keratinocytes [70]. One mechanism by which MMP-2 and MMP-9 activity induce cancer angiogenesis involves the cleavage of latent TGF-β in a CD44-dependent manner that may promote tumor growth and invasion [71]. MMP-9 seems to be strongly associated with angiogenesis. And we also observe the aforementioned effect on HUVECs in conditioned media containing mixtures with specific MMP-9 knockdown (Supplementary Figure S2). Moreover, we observe increased blood flow in perfusion experiments in BMSC/HNSCC co-culture. However, the perfusion measurements were performed with samples that showed only mild leakage because otherwise, the measurements would have proven themselves to be not reliable enough. Most of the mixtures, however, showed relatively strong blood leakage. So we can assume a crossover in which MMP-9 seems to be associated with the increased formation of blood vessels on the one hand, but on the other hand, is also responsible for blood leakage or hemorrhage after prolonged growth. Power et al. observed, among other MMPs, the increase of MMP-9 mRNA in a collagenase-induced intracerebral hemorrhage (ICH) model [72]. Wells and colleagues showed increased levels of MMP-2 and MMP-12 in the collagenase-induced ICH mouse model [73]. Wang et al. demonstrated a deleterious role of MMP-9 in acute brain injury through activation and upregulation of MMP-9 in ICH in the same model using gel zymography [74]. Furthermore, the increased gelatinolytic activity of MMP-2 and MMP-9 was observed in brain ICH in further studies [75,76].

The question of the cellular origin of MMP-9 expression in our 2D and 3D co-culture model initially led us to find that BMSCs were the origin using differential siRNA knockdown [42]. We also examined the regulation of MMP-9 expression in HNSCC cells, as we were interested in whether regulation occurs purely in BMSCs, or whether some form of regulation also occurs on the part of tumor cells. There is new evidence in the literature that mesenchymal stem cells drive metastasis by inducing increased expression and secretion in lung cancer cells [77]. In contrast, Ho et al. have shown that the MMP1 promoter is deregulated in MSCs in a tumor-dependent manner via the SDF-1/CXCR-4 pathway [78].

We found it very intriguing that there seems to be a reciprocal modulatory process, as on the one hand, BMSCs seem to upregulate and secrete active MMP-9, and on the other hand, HNSCC cells seem to simultaneously downregulate MMP-9 promoter activity. One reason for this could be that tumor cells limit their energy consumption and “hijack” stromal cells for their requirements, such as blood supply, clearing the way for invasion. There is also some evidence in the literature describing how cancer cells exploit stromal cell activity to develop adaptive metabolic strategies to promote tumor growth [79,80].

It seems that tumor cells hijack the BMSCs and make them produce MMP-9. Similarly, during melanoma progression, MMP-9 is also activated very early. In this case, not the melanoma cells but neighboring keratinocytes secrete the protease [81]. MMP-9 regulation was shown to be dependent on microenvironmental factors or cells in other studies as well. Van Valckenborgh et al. demonstrated an upregulation of MMP-9 in multiple myeloma cells was caused by the interaction of bone marrow endothelial cells [82]. High variations in the transduction efficiency of MSCs have been observed across species [83]. With increasing plasmid size, integration events decrease further [84]. This might be the reason why we have observed highly inconsistent results by lipofection of BMSCs with the luciferase promoter construct, which did not allow us to make conclusions about MMP-9 promoter activity in BMSCs. Therefore, we decided to demonstrate co-culture-induced expression of MMP-9 in BMSCs via siRNA knockdown experiments, as demonstrated in Wessely et al. [42]. The luciferase promoter construct was efficiently transfected into tumor cells to investigate MMP-9 promoter activity. Since we had already identified BMSCs as being responsible for MMP-9 expression, we did not expect any change in MMP-9 promoter activity in the tumor cells. However, to our surprise, there was a marked degree of regulation of MMP-9 promoter activity. This process seems to be the opposite in adherent 2D and in 3D spheroid cell cultures. In 3D spheroids, BMSCs cause a marked downregulation of MMP-9 promoter activity in tumor cells. In adherent 2D cell culture, co-culture with BMSCs seems to have no or only a weak effect on MMP-9 promoter activity. In the spheroid, tumor cells produce a matrix, potentially causing the cells to interact directly with each other. In the adherent 2D culture, the cells grow side by side on a plastic surface, and the direct mutual effect is thereby not given or attenuated.

Of additional interest, the MMP-9 promoter activity of HNSCC tumor cells in adherent 2D cell culture is fundamentally different from that in 3D spheroid culture. MMP-9 promoter activity in HNSCC cells in 2D co-culture is inversely proportional to cell number, whereas in 3D, MMP-9 promoter activity increases up to 5-fold with higher cell number. Mixing with a consistent count of BMSCs while increasing the number of tumor cells did not have an effect on promoter activity in 2D. However, in 3D, promoter activity in mixtures was significantly enhanced up to 4-fold with increasing tumor cell number. Regarding the amount of used BMSCs, we wanted to analyze the effect on MMP-9 promoter activity with a low-to-medium number of BMSCs, which we have taken from the literature [85,86] and from preliminary data in our lab, where we found 4000 BMSCs with the given numbers of HNSCC cells to be in a good range for cell survival in 3D.

These observations may be considered as a possible background for controversial statements on whether BMSCs promote or inhibit tumors. Depending on the model and the number of cells, depending on whether direct or indirect co-culture is employed, depending on how many cells are used in a particular setting, the extent to which the effect shifts from tumor-promoting to tumor-inhibiting can play a decisive role.

In summary, we found that the direct interaction of HNSCC and BMSCs promotes the tumorigenic properties of BMSCs by inducing MMP-9 expression in direct co-culture. This induction occurs in the BMSCs, while at the same time, tumor cells appear to attenuate their MMP-9 promoter activity. This highlights the potential negative role of BMSCs in recruitment to the tumor region and also demonstrates the mutual influence of two cell types. The interaction of BMSCs and HNSCC cells interferes with the metastatic process, thereby accelerating HNSCC progression. Our findings provide new insights into the complex interaction between HNSCC and its tumor stroma and help to address this in the development of new therapeutics. In future studies, we will explore the mechanisms behind MMP-9 release and hemorrhage upon stromal contact with HNSCC cells.

## 4. Materials and Methods

### 4.1. Cells and Culture Conditions

#### 4.1.1. HNSCC Cells

The human head and neck cancer cell lines SCC-9 and SCC-15 were ordered from ATCC (Manassas, VA, USA) as Head and Neck Panel TCP-1012. The tumor cell lines PCI 1, PCI 13, and PCI 68 were isolated and established at the Pittsburgh Cancer Institute (PCI; Pittsburgh, PA, USA) from squamous cell carcinomas of the head and neck [87,88]. All cell lines were tested using STR DNA typing. Information on the original localization, tumor stage, age and sex of the patients can be found in Table 1.

Tumor cell lines were maintained in DMEM (PanBiotech, Aidenbach, Germany) supplemented with 10% fetal calf serum (FCS, Gibco, Carlsbad, CA, USA), 1% L-glutamine (Sigma-Aldrich, Munich, Germany) and 1% penicillin/streptomycin (Sigma-Aldrich) at 37 °C in a 5% CO_2_ humidified atmosphere. For co-culture experiments, tumor cell lines were adapted to StemMACS Expansion Medium (Miltenyi Biotec GmbH, Bergisch-Gladbach, Germany). The medium was changed every 2–3 days. Cells were passaged prior to reaching confluence. Cell detachment was achieved by incubation with 0.05% trypsin-EDTA solution (Sigma-Aldrich) for 5–10 min (min) at 37 °C.

#### 4.1.2. BMSCs

Human bone marrow-derived mesenchymal stromal cells (BMSCs) were provided by the Department of Experimental Orthopedics (Center for Medical Biotechnology, Asklepios Klinik Bad Abbach, Bad Abbach, Germany). The use of human material followed written informed consent from the patients and was approved by an ethics committee. Bone marrow from 4 different donors was obtained from the caput femoris and acetabulum and was purified by density gradient centrifugation (Table 2) [89]. BMSCs, either in mono-culture or in co-culture, were cultivated in StemMACS Expansion Medium (Miltenyi Biotec GmbH), including 1% penicillin/streptomycin (Sigma-Aldrich). BMSCs were not cultured further until they reached passage 5. All used cells were tested for the BMSCs-associated markers CD73+, CD90+, CD105+, CD19−, and CD34− by flow cytometry to check for contamination with other cell types and the differentiation status of BMSCs.

#### 4.1.3. 2D Adherent Cell Cultivatio

For in vitro 2D cell culture experiments, cell numbers from a total of 25,000 to 105,000 cells were seeded per 6-well (Greiner Bio-One, Kremsmünster, AUT), resulting in a confluence of approximately 50–100% at harvest time. When co-cultured in 2D, cell numbers of BMSCs were maintained at 15,000 cells per 6-well, with cell numbers of HNSCC cells varying from 10,000 to 90,000 cells per well. Thus, the ratio between HNSCC cells and BMSCs, as well as the cell confluence, was varied. The total cell numbers in co-cultivation were the same as those in mono-culture of HNSCC cells to allow direct comparison of the mixing ratio with identical confluence.

#### 4.1.4. 3D Tumor Spheroids Formation

3D tumor cell spheroids were generated by the so-called hanging drop method [90]. For this purpose, a defined number of single detached cells was transferred in the appropriate 3D cultivation medium containing 2% FCS (Gibco), 1% L-glutamine (200 mM) (Sigma-Aldrich), 1% penicillin/streptomycin (Sigma-Aldrich), 1.2% methylcellulose (20% methocel stock solution (Sigma-AldrichChemie GmbH, Taufkirchen, Germany. Cells of a defined cell number were pipetted in a total volume of 30 µL as single drops onto the bottom of the lid of a Petri dish (Greiner Bio-One, Kremsmünster, AUT). Due to the interaction of surface tension and viscosity, hanging drops are formed, which, together with the lack of adhesion to a surface, enable cells to grow in a spheroid 3D structure. To create a humid atmosphere, the bottom of the Petri dish was filled with 5 mL of phosphate-buffered saline (PBS) (Sigma-Aldrich Chemie GmbH, Taufkirchen, Germany). The lid was then carefully placed on the Petri dish, and the hanging drops were cultured at 37 °C in a 5% CO_2_ humidified atmosphere for 24 h.

In vitro spheroid populations were generated with cell numbers ranging from 7000 to 27,000 cells per spheroid. The different spheroid sizes resulted in varying degrees of hypoxic effects on the cells. When co-cultured, the cell number of BMSCs was maintained at 4000 cells per spheroid, with HNSCC cells varying from 3000 to 23,000 cells per spheroid. The total cell numbers in co-cultivation were the same as those in mono-culture of HNSCC cells in 3D to allow direct comparison of the mixing ratio with corresponding spheroid size.

### 4.2. Chorioallantoic Membrane (CAM) Model

For in vivo experiments, the chorioallantoic membrane (CAM) model was used, as previously described in Kuri et al. [53] and Kohl et al. [91]. Fertilized chicken eggs were incubated in a ProCon egg incubator (Grumbach, Aßlar, Germany) at a constant temperature of 37.8 °C and 63% humidity with occasional rotation.

After the first three days of incubation, a hole was carefully cut in the eggshell without damaging the CAM. The hole was then sealed with tape. Eggs were incubated for additional 4 days without rotation until the day of inoculation on day 7. For inoculation on day 7, either BMSCs or HNSCC in mono-culture or co-cultures were used for the experiments. For mono-culture and co-culture inoculation, 2 × 10^6^ cells were embedded in 30 μL Corning^®^ Matrigel^®^ (Thermo Fisher Scientific GmbH, Waltham, MA, USA) and placed in the center of an agarose ring (1% agarose in ddH_2_O, Sigma-Aldrich) on the CAM. The first perfusion measurement was performed on day 8. The experiment ended on day 14 after the second perfusion measurement.

Cells were treated with 10 µM of the specific MMP-9 inhibitor JNJ0966 (Biozol, Eching, Germany) on day 1 and day 3. To keep the reagent in place, 1.2% methylcellulose (Sigma-Aldich) was added to the inhibitor solution to increase viscosity. The control treatments each contained 0.1% DMSO (Sigma-Aldrich), which was equivalent to the DMSO concentration of the inhibitor solution.

### 4.3. Assays and Methods

#### 4.3.1. Luciferase Activity Assay

A luciferase activity assay was performed to determine MMP-9 promoter activity. The assay was performed using the Dual-Luciferase^®^ Reporter Assay (Promega, Madison, WI, USA). The Firefly luciferase gene was linked to the promoter sequence of interest to measure its activation using a luminometer (Luminex, Austin, TX, USA) [60]. The MMP-9 promoter element was cloned into vector pGL4.16 (luc2/Neo), which contains the Firefly luciferase gene (PromegaMadison, WI, USA). The MMP-9 promoter used was 930 base pairs in size with the coding regions:

5′-TACATTGGTACCTCTTGGGTCTTGGCCTTAGT-3′ and

5′-TTGATACTCGAGCCAGCACCAGGAGCACC-3′.

Adherent tumor cells cultivated in 2D were washed twice with PBS (Sigma-Aldrich) and lysed afterward with 300 μL PLB (passive lysis buffer) for 15–20 min with gentle agitation. For the measurement, 50 μL of cell lysate was used for each sample.

For the analysis of cells from 3D cultivation, 30 spheroids were collected in a 1.5 mL microcentrifuge tube (Eppendorf AG, Hamburg, Germany). This was followed by two washing steps with 700 μL PBS. Subsequently, 100 μL PLB lysis buffer was added to the spheroids. The spheroids of all tumor cell lines used were put on a shaker for 15 min for to optimize lysis. Afterward, the tumor cells were exposed to two additional freeze and thaw cycles in liquid nitrogen. For measurement, 50 μL of cell lysate was used per sample.

As controls, we used cells that were not transfected or cells that were treated with transfection reagents only. To compensate for differences in transfection efficiency and cultivation-related variations and allow for comparability of measurements, as a control vector, co-transfection was performed with pGL4.74 (hRluc/TK; PromegaMadison, WI, USA). The signal generated by the co-vector correlates with the amount of vector transfected and, thus, the transfection efficiency (https://www.promega.com/-/media/files/resources/paguide/letter/chap8.pdf?la=en, accessed on 23 November 2022). Measurements were taken in relative light units (RLU; Lumat LB 9507, Berthold Technologies, Oak Ridge, TN, USA), which correlated with luciferase activity.

#### 4.3.2. Gelatin Zymography

Zymography is a variant of classical SDS-PAGE whereby enzymes can be digested by adding a substrate in an acrylamide gel, in this case, gelatin. In addition to active forms on MMPs, all other isoforms can also be visualized in zymography. The latent form of the enzyme becomes active by denaturation and renaturation and can thus be measured. Enzyme amounts as little as picograms (pg) are detectable in zymography [92]. Here, zymography was used to detect the protein expression of MMP-9 and MMP-2.

Serum-free cell culture supernatants were used for the analysis of MMP activity. To exclude the serum, spheroids growing in hanging drops were collected after 2 d of cultivation, washed in 1 mL of PBS, and reseeded for another 24 h in serum-free media in a 24-well plate (Greiner Bio-One). The protein concentration of the supernatant was determined using the Pierce Bicinchoninic Acid Protein (BCA)-Assay (Thermo Fisher ScientificWaltham, MA, USA). Two micrograms of total protein per lane were applied for zymography in the loading buffer.

SDS-PAGE was performed in a 10% acrylamide gel, including 0.1% gelatin (Sigma-Aldrich). Proteins were separated based on their molecular weight using a current of 20 mA. During the separation, the gel chamber was cooled with ice to prevent the gel from melting due to the generated heat. Separation took a total of 5 h. Proteins with a molecular weight lower than 50 kDa were intentionally lost during the separation to achieve better resolution of bands above 50 kDa.

To restore the tertiary structure of the proteins, the gel was then incubated twice for 15 min in a renaturation buffer containing 2.5% TritonX (Sigma-Aldrich) since the native state of the protein was essential for the digestion of the gelatinases. This was followed by 4 washing steps for 5 min, each in ddH_2_O. Digestion of gelatinase in the gels by MMPs was performed at 37 °C in 20 mL digestion buffer, containing 50 mM Tris-HCl (Carl Roth GmbH + Co. KG, Karlsruhe, D) pH 8.5 and 5 mM CaCl (Sigma-Aldrich), for a total of 36 h. Afterward, the gels were stained with Coomassie Blue staining solution for 1 h. To remove the color from the digested bands, decolorization was then performed in decolorizing solution I, containing 50% Methanol (Supelco, Bellefonte, PA, USA) and 10% acetic acid (Carl Roth), for 1 h and in decolorizing solution II (5% methanol and 7.5% acetic acid) for 4 h. The gel was imaged using Gel Doc EZ Imager (Bio-Rad, Hercules, CA, USA).

#### 4.3.3. Transient MMP-9 siRNA Knockdown

To further investigate MMP-9 activity in BMSCs, siRNA knockdown of MMP-9 was performed. Non-targeting siRNA served as control. Transfection was performed on adherent cells. Therefore, a cell number of 160,000 BMSCs was seeded in a T25 cell culture flask (Corning Inc., Corning, NY, USA) two days before transfection.

For transfection, 22.5 µL of RNAiMAX (Thermo Fisher Scientific, Waltham, MA, USA) transfection reagent was diluted with 375 µL of Gibco Opti-MEM I Reduced Serum Media (Thermo Fisher Scientific). In a separate tube, 6.25 µL siRNA with a stock concentration of 20 µM (MMP-9 siRNA or non-targeting siRNA) was diluted with 375 µL Gibco Opti-MEM I Reduced Serum Media. For the formation of reaction complexes of siRNA and transfection reagent, both were mixed and incubated at RT for 5 min. To prepare the cells for transfection, the regular stem cell culture medium in the T25 cell culture flasks was replaced with 5 mL Gibco Opti-MEM I Reduced Serum Media without antibiotics. For each flask, 750 µL of transfection mix was added to the cells. Cells were used for further analyses 24 h after incubation at 37 °C in a 5% CO_2_ humidified atmosphere.

#### 4.3.4. Tube Formation Assay

For the analysis of mesh formation, 40,000 human umbilical vascular endothelial cells (HUVECs) were seeded in 24-well plates (Greiner Bio-One) coated with 100 µL Matrigel^®^ (8.8 mg/mL; Corning). One ml of conditioned media (CM) from HNSCC mono-cultures, a BMSC/HNSCC co-culture, and a co-culture with prior MMP-9 siRNA knockdown, grown for 24 h at 70–80% confluency was added to HUVECs. After 6 h, mesh formation cells were analyzed using an ImageJ Angiogenesis Analyzer (https://doi.org/10.1038/s41598-020-67289-8, accessed on 23 November 2022).

#### 4.3.5. Angiogenesis Measurement via Laser Speckle Contrast Imaging (LSCI)

The perfusion status of the CAMs was measured on day 8 and 14 using the PeriCam PSI system HR model (Perimed Instruments GmbH, Rommerskirchen, Germany) to obtain semiquantitative data based on LSCI technology. The working distance between the laser head and the surface of the CAM was 100 mm. A sampling frequency of 16 Hz was chosen. Average values were calculated from 32 frames, giving an effective frame rate of 0.5 frames/s. Using a visible laser light of 650 nm, 3 mW, the operator could select the desired area with the tumor in the center. A 785 nm, 80 mW laser was used for the measurement. To obtain accurate results, the embryo had to be still for at least 5 consecutive measurements (10 s). The TOIs (time of interest) were chosen according to the plateaus that occurred once the embryo was static. Semiquantitative units of blood flow and area were averaged over the sampling period during the plateau. Average blood flow was calculated using PimSoft version 1.5 software (Pimsoft, Inc., Torino, ITA). The calibration process is performed with a calibration box containing 2 reference ranges, a 0 perfusion range and a high perfusion range. The high perfusion region is filled with a motility standard consisting of a colloidal suspension of polystyrene particles and has a perfusion value of 250 ± 5 perfusion units (PU).

### 4.4. Statistical Analysis

Statistical analyses were performed using GraphPad Prism 8 software (GraphPad Software, Inc., San Diego, CA, USA). All assays were performed in replicates. The results are presented as means ± SD. A 1-way ANOVA was used to compare multiple groups. Where necessary, 2 groups were compared with a 2-tailed Student’s *t*-test. *p* ≤ 0.05 was considered statistically significant.

## Figures and Tables

**Table 1:** Tumor cell line origin, m = male, n.a. = not available.

Cell Line	Origin	TNM Status	Type of Tumor	Age, Sex
SCC-9	tongue	n.a.	primary tumor	25, m
SCC-15	tongue	n.a.	primary tumor	55, m
PCI 1	larynx	pT2N0M0G2	primary tumor recurrence	65, m
PCI 13	retromolar area	pT4N1M0G3	primary tumor	50, m
PCI 68	tongue	pT4N0M0G1	n.a.	n.a.

**Table 2:** BMSC origin, m = male, f = female.

Donor Number	Age, Sex	Origin
706	67, m	Acetabulum
821	60, f	Acetabulum
822	84, f	Acetabulum
866	63, f	Caput femoris

## Data Availability

Not applicable.

## Supplementary Materials

The following supporting information can be downloaded at: https://www.mdpi.com/article/10.3390/ijms24021293/s1.
