# Supplementary figures and images for: Opposing MMP-9 Expression in Mesenchymal Stromal Cells and Head and Neck Tumor Cells after Direct 2D and 3D Co-Culture
Source: Int J Mol Sci. 2023 Jan 9;24(2):1293. doi: 10.3390/ijms24021293 (PMC9861345; doi:10.3390/ijms24021293)

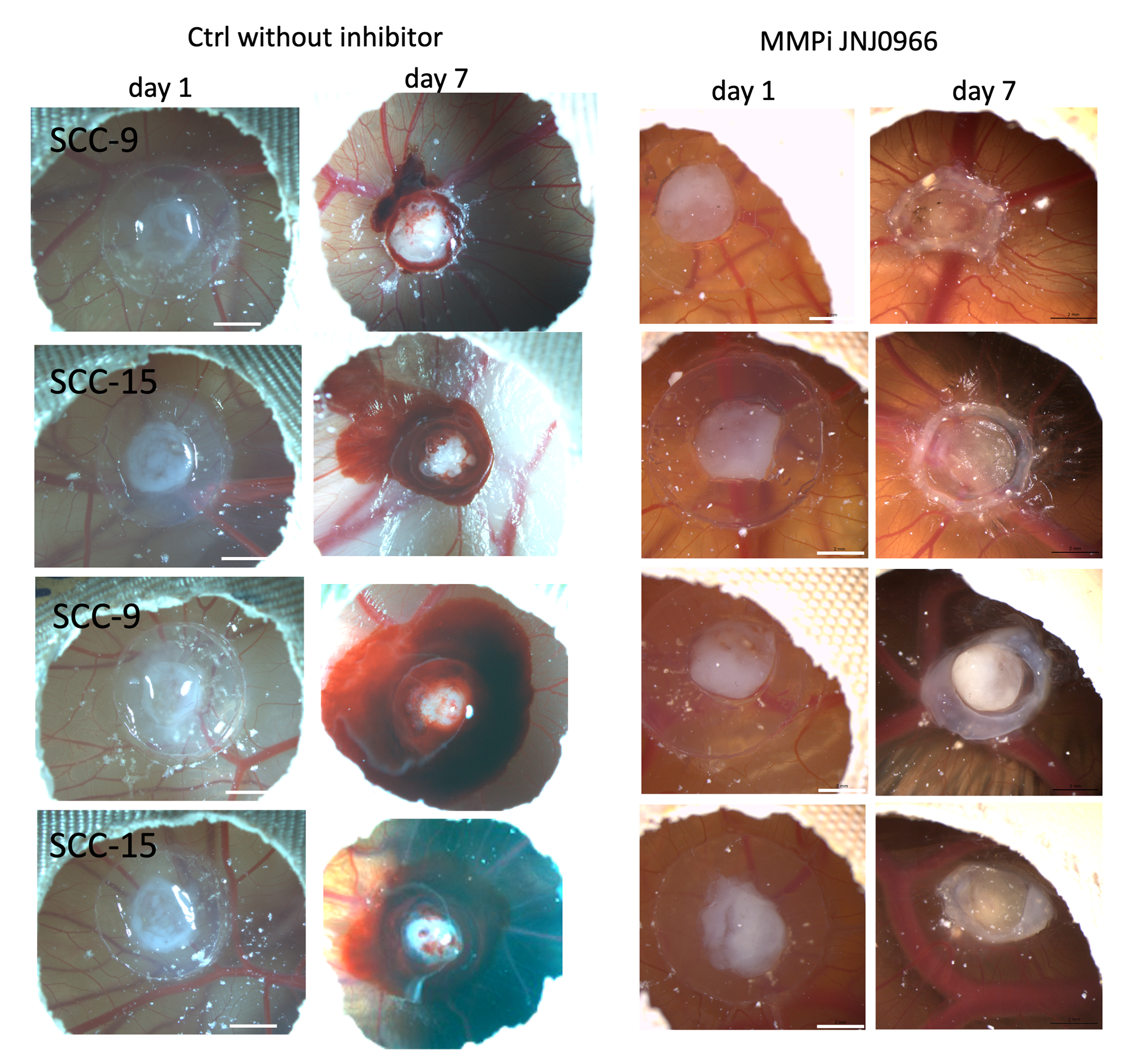

Supplement: Supplementary file 1 [file ijms-24-01293-s001.zip › suppl figure S1.tif]

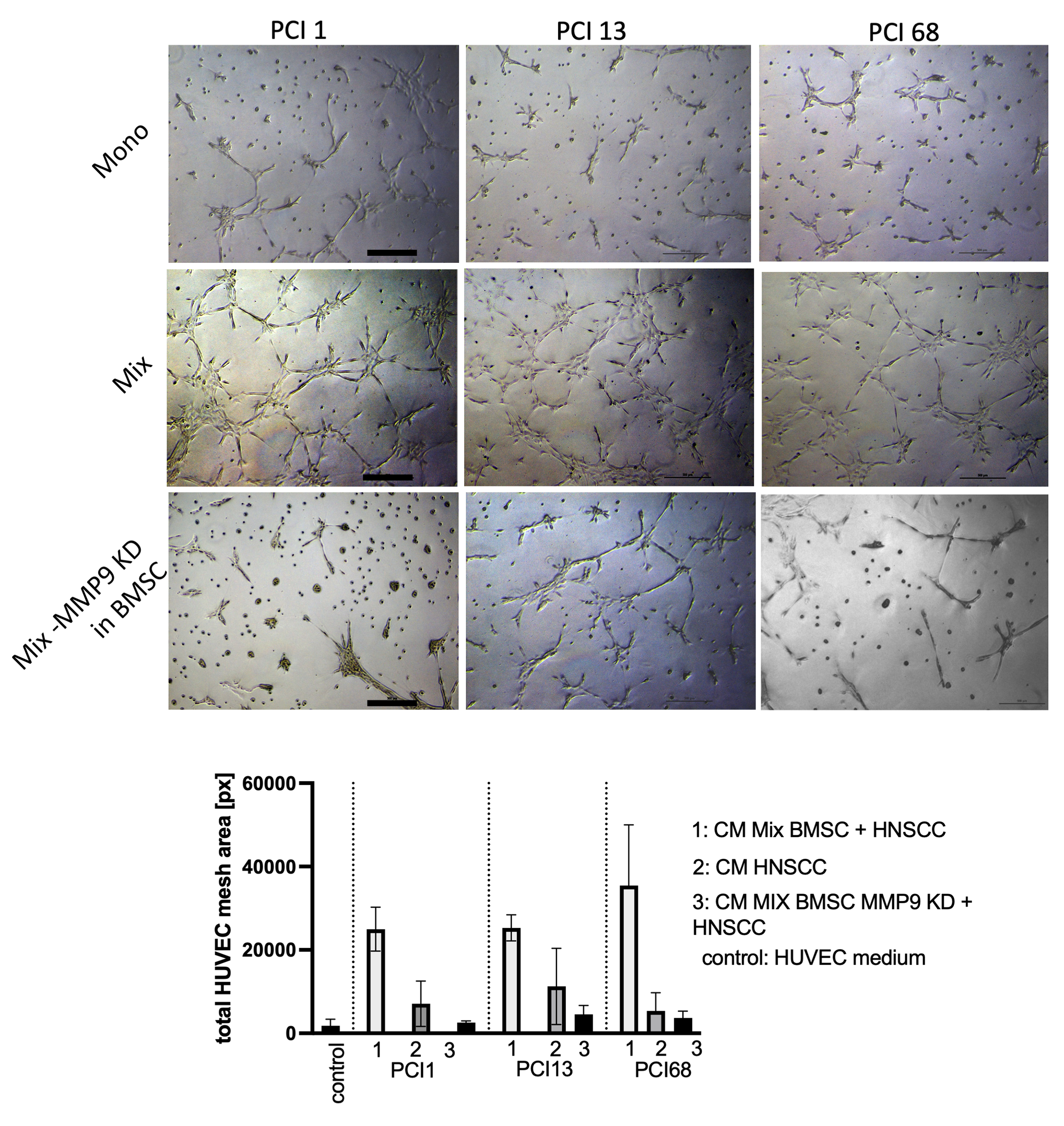

Supplement: Supplementary file 1 [file ijms-24-01293-s001.zip › suppl figure S2.tif]
